# Risk factors for identifying pulmonary aspergillosis in pediatric patients

**DOI:** 10.3389/fcimb.2025.1616773

**Published:** 2025-06-27

**Authors:** Shangmin Yang, Yanmeng Sun, Mengyuan Wang, Huan Xu, Shifu Wang

**Affiliations:** ^1^ Department of Microbiology Laboratory, Children’s Hospital Affiliated to Shandong University (Jinan Children’s Hospital), Jinan, China; ^2^ Department of Clinical Microbiology, Shandong Provincial Clinical Research Center for Children’s Health and Disease, Jinan, China; ^3^ Department of Scientific Affairs, Vision Medicals Center for Infectious Diseases, Guangzhou, China

**Keywords:** pulmonary aspergillosis, metagenomic next-generation sequencing, ROC curve, risk factors, pediatrics

## Abstract

**Objectives:**

This study aimed to identify the independent risk factors and develop a predictive model for pulmonary aspergillosis (PA) in pediatric populations.

**Methods:**

This retrospective study compromised 97 pediatric patients with pulmonary infections (38 PA cases and 59 non-PA cases) at Children’s Hospital Affiliated to Shandong University between January 2020 and October 2024. Multivariate binary logistic regression was used to identify PA-associated risk factors. Receiver operating characteristic (ROC) curves, calibration plots, and Brier scoring were used to evaluate the diagnostic model.

**Results:**

8 clinical variables significantly differed between the PA and non-PA groups. Multivariate binary logistic regression analysis identified six significant independent risk factors: a history of surgery (OR: 9.52; 95% CI: 1.96–46.23; *P* = 0.005), hematologic diseases (OR: 11.68; 95% CI: 0.89–153.62; *P* = 0.062), absence of fever (OR: 8.244; 95% CI: 1.84–36.932; *P* = 0.006), viral coinfection (OR: 15.99; 95% CI: 3.55–72.00; *P* < 0.001), elevated (1, 3) -β -D-glucan levels (BDG, > 61.28 pg/mL; OR: 7.38; 95% CI: 1.26–43.31; *P* = 0.027), and shorter symptom-to-admission interval (< 4.5 days; OR: 38.68; 95% CI: 5.38–277.94; *P* < 0.001) were risk factors for PA. The predictive model demonstrated excellent discrimination (AUC 0.93, 95% CI 0.88-0.98) and calibration (Hosmer-Lemeshow p=0.606, R²=0.96, Brier score 0.097). metagenomic next - generation sequencing (mNGS) revealed significantly higher rates of polymicrobial infections in PA cases (86.84% vs 18.64%, p<0.001).

**Conclusions:**

This study established and validated a high-performance predictive model incorporating six clinically accessible parameters for the diagnosis of pediatric PA.

## 1 Introduction

*Aspergillus*, a ubiquitous opportunistic filamentous fungus, primarily invades the human host through the respiratory tract, potentially leading to a spectrum of pulmonary pathologies collectively termed pulmonary aspergillosis (PA). PA predominantly affects immunocompromised patients (Kanj et al., 2018), with particularly high morbidity in pediatric populations due to immature immune system and frequent comorbidities (notably hematologic malignancies and primary immunodeficiencies) (Wattier et al., 2015). Epidemiological data from the European Registry of Invasive Fungal Infections in Children (ECIFIG) reveal a pediatric incidence rate of invasive PA at 1.2 cases per 100,000 population. Notably, pediatric mortality rates (40%–60%) significantly exceed those observed in adult populations (Groll et al., 2014). The risk escalates dramatically in high-risk cohorts, particularly hematopoietic stem cell transplant recipients and chemotherapy patients, where the incidence of PA reaches 15–20% (Zaoutis et al., 2006).

The diagnosis of PA in pediatric populations remains clinically challenging due to three limitations: (1) non-specific clinical presentation (e.g., fever, cough, dyspnea) that overlap with common respiratory infections; (2) technical difficulties in obtaining adequate lower respiratory tract specimens, particularly bronchoalveolar lavage fluid (BALF), result in suboptimal sensitivity (<50%) when using conventional microbiological diagnostic methods (Dinand et al., 2016); (3) lack of standardized diagnostic thresholds for serological biomarkers, particularly galactomannan (GM) assays in pediatric population (Warris et al., 2019). Consequently, these diagnostic constraints highlight the urgent need for the identification of pediatric-specific risk factors and the establishment of predictive models for pediatric PA to improve early detection and clinical outcomes.

In recent years, research on risk factors for PA in pediatric populations has gained increasing attention. While classical predisposing factors—neutropenia and glucocorticoid therapy—remain clinically significant, emerging evidence underscores unique pediatric risk factors, including a history of preterm birth, congenital heart disease requiring surgical intervention, and Epstein-Barr virus coinfection (Wattier et al., 2015). Notably, developmental immunometabolism features in children may exacerbate disease progression, for example, heightened iron metabolism could facilitate *Aspergillus fumigatus* iron acquisition pathways (Schrettl and Haas, 2011). Despite these insights, current research remains disproportionately focused on adult populations, resulting in critical knowledge gaps regarding pediatric-specific risk stratification, validated biomarker thresholds, and multifactorial pathogenic interactions. These limitations significantly hinder the development of precision diagnostic and therapeutic frameworks for PA in children. Thus, we performed a comprehensive analysis of PA risk factors in pediatric patients through a retrospective case-control study encompassing 97 pulmonary infections (38 PA cases vs 59 controls) in this study.

## 2 Materials and methods

### 2.1 Study design and participants

The diagnostic criteria of PA followed by the expert group are mainly based on international diagnostic criteria, including the IDSA and ESCMID-ECMM-ERS guidelines (Warris et al., 2019) proposed by the experts. Among them, microbiological evidence based on histopathology/cytopathology or sterile site culture is used as the criterion for diagnosis as proven PA, and the combination of host factors, clinical/imaging features and microbiological/molecular biological markers is used as the criterion of the clinical diagnostic criteria as probable PA. A total of 97 pediatric pneumonia were retrospectively enrolled between January 2020 and October 2024 at Children’s Hospital Affiliated to Shandong University, among which 38 was PA (15 cases were diagnosed as proven PA and 23 cases were probable PA). Patients enrolled in this study should meet the following inclusive criteria: (1) age<18; (2) diagnosed with pneumonia meeting the guidelines for the management of community-acquired pneumonia in children (Subspecialty Group of Respiratory, t.S.o.P.C.M.A et al., 2024); (3) obtaining respiratory species; (4) having metagenomic next - generation sequencing (mNGS) results. The diagnostic criteria for the PA were as follows (Society of Pediatrics, C.M.A and Editorial Board, C.J.o.P, 2022): 1. Clinical Manifestations: Predisposing conditions include primary or secondary immunodeficiency, chronic underlying diseases, or long-term indwelling internal catheters. Common symptoms include fever, cough, and wheezing. Severe cases may present chest pain and hemoptysis. Extrapulmonary manifestations, such as sinusitis or nasal bone destruction, may occur. 2. Imaging Findings: Radiographic features are generally typical non-specific, including infiltrating lamellar opacities or atelectasis. Specific signs, such as the halo sign, tree-in-bud sign, or wedge-shaped infarcts, may be observed on computed tomography (CT). 3. Laboratory and Pathological Examination: Direct microscopy of sputum or BALF reveals mycelium elements, with or without positive fungal culture result. Serological markers, such as galactomannan (GM) or (1, 3) -β -D-glucan (BDG), are positive. Histopathological examination of lung tissue reveals Aspergillus infection with mycelial presence. Molecular test, including polymerase chain reaction (PCR) or mNGS, confirms Aspergillus in tissue or BALF samples. The clinical diagnosis for both PA and non-PA cases was initially made by two senior respiratory specialists, based on a comprehensive evaluation of clinical symptoms, laboratory test results, chest computed tomography (CT) imaging, mNGS etiology, and clinical responses to treatment. To ensure diagnostic consistency and accuracy, any discrepancies in the initial diagnoses were resolved through a consensus process involving a unified expert panel. This panel, comprising three senior respiratory specialists, conducted a detailed review of all cases with conflicting diagnoses. The panel’s assessment included a thorough evaluation of the patients’ clinical presentations, radiological findings, laboratory data, microbiological results, and treatment outcomes. The final diagnosis was determined based on the consensus reached by this expert panel, thereby minimizing potential variability in judgments and ensuring a unified and reliable diagnostic outcome. Clinical data—including sex, age, underlying conditions, clinical presentations, CT imaging, mNGS tests, and laboratory results—were collected from the patients’ medical histories.

This study was approved by the Ethics Committee of the Children’s Hospital Affiliated to Shandong University (No.: SDFEEB/P-2022017) and conducted in accordance with the Declaration of Helsinki.

### 2.2 Metagenomic next-generation sequencing

The DNA was extracted from BALF using a QIAamp ^®^ UCP Pathogen DNA Kit (Qiagen), adhering to the manufacturer’s instructions. Human DNA was removed using Benzonase (Qiagen) and Tween 20 (Sigma). Total RNA was extracted with a QIAamp ^®^ Viral RNA Kit (Qiagen) and ribosomal RNA was removed with a Ribo-Zero rRNA Removal Kit (Illumina). cDNA was generated using reverse transcriptase and dNTPs (Thermos Fisher). Libraries were constructed for the DNA and cDNA samples using a NextEra XT DNA Library Prep Kit (Illumina, San Diego, CA). The library was purified, and magnetic beads selected the fragments. The library quality was assessed with a Qubit dsDNA HS Assay Kit followed by a High Sensitivity DNA kit (Agilent) on an Agilent 2100 Bioanalyzer. The library pools were then loaded onto an Illumina NextSeq CN500 sequencer for 75 cycles of single-end sequencing to generate approximately 20 million reads for each library. For negative controls, we also prepared sterile deionized water in parallel with each batch to serve as a non-template control, using the same protocol.

High-quality sequencing data were generated by removing low quality and short (length < 40 bp) reads, followed by computational subtraction of human host sequences mapped to the human reference genome (hg38 and YH sequences) using Burrows-Wheeler alignment. The remaining data obtained by removing low-complexity reads were classified by simultaneous alignment to four microbial genome databases, consisting of viruses, bacteria, fungi, and parasites. The classification reference databases were downloaded and optimized from public databases such as NCBI and GenBank. In the end, the multi-parameters of Species in the microbial genome databases were calculated and exported, and professionals with microbiology and clinical backgrounds interpreted the results.

### 2.3 Statistical analysis

The data were analyzed using IBM SPSS statistical software (version 26.0) and R software (version 4.4.1). Continuous variables were expressed as median (interquartile range, IQR) or mean (standard deviation, SD), and categorical variables as frequencies (%). Group comparisons employed Mann-Whitney U tests (non-normal data) or Student’s *t*-tests (normal data) for continuous variables, and chi-square or Fisher’s exact tests for categorical variables. Univariate logistic regression was used to identify the risk factors associated with PA. Variables with P-value < 0.1 were further analyzed via multiple logistic regression to construct the diagnostic model. The diagnostic model was evaluated using receiver operating characteristic (ROC) curves and Hosmer-Lemeshow goodness-of-fit tests (Nahm, 2022). ROC curves and calibration plot were produced utilizing GraphPad Prism (version 9.4.1). And the forest plots were generated using the R forestploter package (version 1.1.2).

### 2.4 Diagnostic model development

Significantly different variables in univariate analysis were incorporated into a multivariate logistic regression model. Odds ratios (ORs) with 95% confidence intervals (CIs) were computed to evaluate risk factor associations. Internal validation utilized bootstrapping (1,000 resamples), and predictive performance was assessed via the Brier score and ROC-derived AUC, following best practices for clinical prediction models (Zhang et al., 2024).

## 3 Results

### 3.1 Patient characteristics

The study enrolled 97 pediatric patients with pneumonia, including 38 cases (39.2%) confirmed with *Aspergillus* spp. infection (PA group) and 59 controls (non-PA group). The cohort consisted of 63 male patients (64.9%) with a median age of 5.33 years (interquartile range [IQR] 2–7). As shown in **Table 1**, eight variables exhibited statistically significant differences between the groups. Biochemical analysis indicated higher levels of hemoglobin (106.5 vs. 120 g/L; *P* = 0.014), procalcitonin (PCT; 0.14 vs. 0.09 ng/mL; p=0.042), and BDG (37.5 vs. 37.5 pg/mL; *P* = 0.022) in PA group. The PA cohort showed greater surgical history prevalence (31.85% vs. 8.47%; *P* = 0.0035), while the non-PA group exhibited higher fever incidence (61.02% vs. 18.42%; *P* < 0.001). Notably, viral co-infection rates (50.00% vs. 16.75%; *P* < 0.001) and median length of hospital stay (19 vs. 11 days; *P* < 0.001) were significantly increased in PA patients. CT imaging revealed greater absence of pulmonary edema prevalence among non-PA subjects (44.07% vs. 23.68%; *P* = 0.043).

**Table 1:** Patient’ characteristics, laboratory findings and CT imaging of PA and non-PA pediatric patients.

Variants	Total (n=97)	non-PA (n=59)	PA (n=38)	P value
**Baseline factors**				
Age, median (IQR) (year)	5.33 (2,7)	5.83 (2.13,7.5)	4.58 (1.62,7)	0.6331
Gender (male), n (%)	63 (64.95)	41 (69.49)	22 (57.89)	0.2426
Clinical manifestations				
Fever, n (%)	43 (44.33)	36 (61.02)	7 (18.42)	< 0.001
Symptom-to-admission, median (IQR)	11 (5,20)	14 (7,20)	10 (4,19.75)	0.0656
Viral coinfection, n (%)	29 (29.9)	10 (16.95)	19 (50)	< 0.001
Co-infected Mycoplasma, n (%)	43 (44.33)	27 (45.76)	16 (42.11)	0.7234
Underlying diseases				
Surgical history, n (%)	17 (17.53)	5 (8.47)	12 (31.58)	0.0035
Diabetes, n (%)	1 (1.03)	0 (0)	1 (2.63)	0.3918
Hematologic diseases, n (%)	8 (8.25)	2 (3.39)	6 (15.79)	0.0535
Premature birth, n (%)	7 (7.22)	4 (6.78)	3 (7.89)	1
VLBW, n (%)	2 (2.06)	2 (3.39)	0 (0)	0.5185
Hormone use, n (%)	69 (71.13)	46 (77.97)	23 (60.53)	0.0643
Laboratory findings				
G, median (IQR) (pg/mL)	37.5 (37.5,43.92)	37.5 (37.5,37.5)	37.5 (37.5,83.96)	0.0223
GM, median (IQR) (μg/L)	0.12 (0.09,0.16)	0.12 (0.09,0.15)	0.12 (0.09,0.21)	0.6573
WBC, median (IQR) (10^9^/L)	9.27 (6.71,12.5)	9.41 (6.98,12.27)	8.97 (5.66,12.46)	0.6953
N%, mean (SD)	56.27 (18.91)	54.73 (19.04)	58.65 (18.71)	0.3216
L%, mean (SD)	34.99 (18.2)	36.51 (17.99)	32.64 (18.51)	0.3094
EO%, median (IQR)	0.4 (0.1,1.8)	0.6 (0.15,1.85)	0.25 (0,1.05)	0.0513
HB, median (IQR) (g/L)	117 (105,128)	120 (113,128)	106.5 (96,127)	0.014
PLT, median (IQR) (10^9^/L)	352 (270,454)	373 (284.5,453.5)	336 (214,453.5)	0.2505
ESR, median (IQR) (mm/h)	23 (12,38)	23 (8,38.5)	26 (15,33)	0.3437
CRP, median (IQR) (mg/L)	4.53 (0.5,19.66)	3.47 (0.5,16.3)	6.59 (1.92,29.98)	0.165
PCT, median (IQR) (ng/L)	0.1 (0.06,0.22)	0.09 (0.05,0.18)	0.14 (0.07,0.33)	0.042
Alb, median (IQR) (g/L)	37.5 (34,40.3)	37.5 (35.1,40.05)	37.45 (33.4,40.38)	0.542
AST, median (IQR) (U/L)	33 (26,43)	34 (26.5,43)	31 (24.25,41.75)	0.4331
ALT, median (IQR) (U/L)	20 (13,42)	20 (13.5,43)	17 (13,40.75)	0.4572
LDH, median (IQR) (U/L)	277 (223,368)	286 (227,366)	266 (208.25,387)	0.5396
Creatinine, mean (SD) (μmol/L)	26.82 (8.8)	27.64 (7.59)	25.55 (10.37)	0.2551
Urea, median (IQR) (mmol/L)	3.8 (2.85,4.7)	3.7 (2.74,4.4)	3.81 (3.12,4.81)	0.2312
DB, median (IQR) (μmol/L)	2.9 (2.3,4.3)	2.9 (2.3,4.1)	2.9 (2.2,5)	0.8158
TB, median (IQR) (μmol/L)	6.3 (4.7,10.3)	5.7 (4.55,8.4)	7.25 (4.73,12.75)	0.1658
TP, mean (SD) (μmol/L)	63.82 (7.5)	63.79 (7.73)	63.88 (7.24)	0.9504
D-dimer, median (IQR) (mg/L)	0.84 (0.41,2.06)	0.78 (0.4,1.92)	1 (0.43,2.26)	0.4872
ATT, median (IQR) (s)	27.6 (24.4,31)	27.6 (24.05,31.4)	27.45 (25.18,30.98)	0.7394
PT, median (IQR) (s)	12 (11.2,13)	11.9 (11.25,12.95)	12.2 (10.95,13.07)	0.7589
CD8%, median (IQR)	27.01 (24.11,31.56)	27.89 (24.58,35.34)	26.13 (23.15,28.88)	0.1454
CD4%, mean (SD)	32.88 (9.19)	33.42 (9.11)	32.04 (9.38)	0.4738
CD3%, median (IQR)	66.54 (57.23,73.63)	66.63 (60.55,73.66)	63.87 (53.9,72.07)	0.1933
Culture, n (%)	19 (19.59)	10 (16.95)	9 (23.68)	0.4146
Computed Tomography images				
Consolidation, n (%)	79 (81.44)	45 (76.27)	34 (89.47)	0.1025
GGO, n (%)	4 (4.12)	1 (1.69)	3 (7.89)	0.2963
Patchy shadow, n (%)	90 (92.78)	53 (89.83)	37 (97.37)	0.2404
Pulmonary nodule, n (%)	11 (11.34)	5 (8.47)	6 (15.79)	0.3314
Pleural effusion, n (%)	45 (46.39)	24 (40.68)	21 (55.26)	0.1597
Emphysema, n (%)	35 (36.08)	26 (44.07)	9 (23.68)	0.0413
Outcome				
Duration of hospital stay (IQR) (day)	12 (10,19)	11 (9,14)	19 (12,22.75)	< 0.001

VLBW, very low birth weight; BDG, (1, 3) -β -D-glucan; GM, Galactomannan; WBC, White blood cell count; N%, Neutrophil percentage; L%, Lymphocyte percentage; EO%, Eosinophil percentage; HB, Hemoglobin; PLT, Platelet count; ESR, Erythrocyte sedimentation rate; CRP, C-reactive protein; PCT, Procalcitonin; Alb, Albumin; AST, Aspartate aminotransferase; ALT, Alanine aminotransferase; LDH, Lactate dehydrogenase; DB, Direct bilirubin; TB, Total bilirubin; TP, Total protein; APTT, activated partial thromboplastin time; PT, Prothrombin time; CD8%, CD8+ T cell percentage; CD4%, CD4+ T cell percentage; CD3%, CD3+ T cell percentage; GGO, ground-glass opacity.

### 3.2 Risk factors for PA

**Figure 1:**
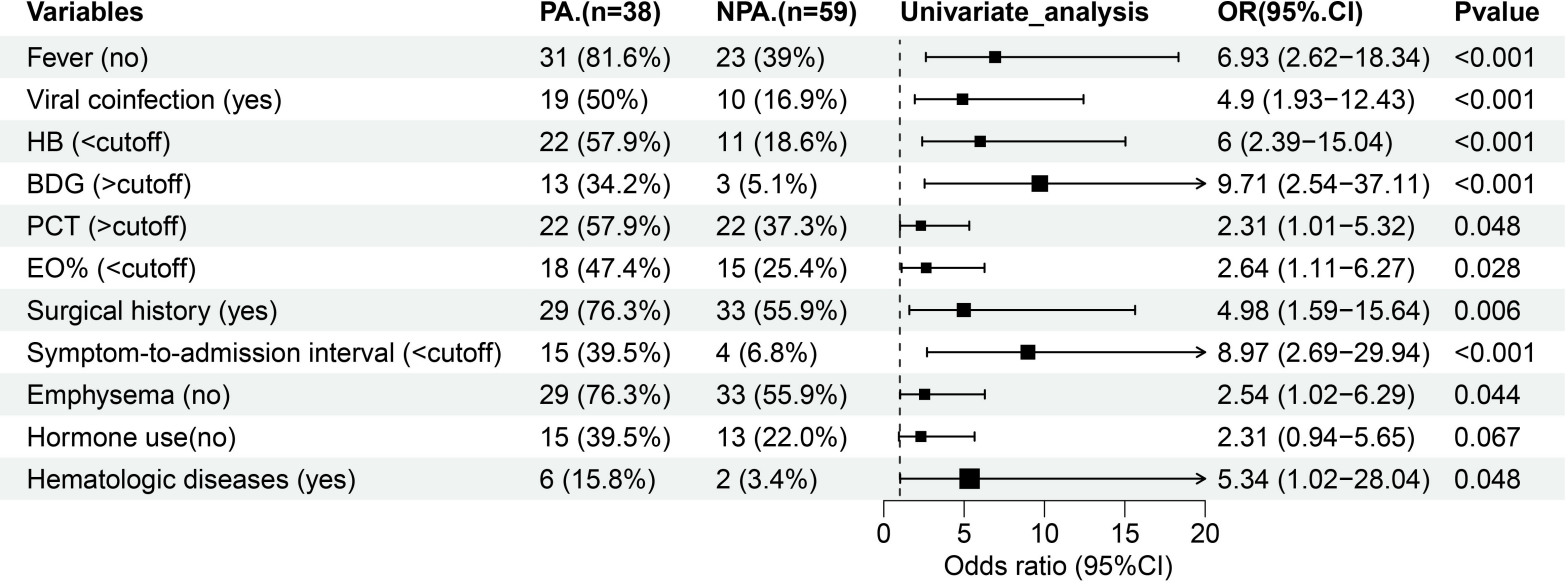
Forest map of 11 risk factors identified in the univariate logistic analysis for the PA group. HB, hemoglobin; BDG, (1, 3) -β -D-glucan; PCT, procalcitonin; EO%, eosinophil percent.

In addition to the eight significantly different variables, four other clinical parameters–eosinophil percent (EO%), symptom-to-admission interval, hematologic diseases, and prior to hormone use–showed different between the groups (*P* < 0.1). To evaluate the predictive capacity of these 12 variables for PA, we performed receiver operating characteristic (ROC) curve analysis and binary logistic regression (**Table 2**). Using the maximum Youden index (Nahm, 2022), the optimal cut-off values for continuous variables were derived from ROC curves. The thresholds were as follows: hemoglobin (111 g/L), BDG (61.275 pg/mL), PCT (0.1045 ng/mL), EO% (0.15), and symptom-to-admission interval (4.5 days). Among the analyzed variables, nine exhibited AUCs > 0.60, indicating moderate predictive utility: absence of fever (AUC: 0.713; 95% CI: 0.608–0.818; *P* < 0.001), viral coinfection (0.665; 0.551–0.780; *P* = 0.006), hemoglobin (0.6485; 0.524–0.7731; *P* = 0.014), BDG (0.6249; 0.5131–0.7367; *P* = 0.038), PCT (0.6229; 0.5086–0.7372; *P* = 0.042), EO% (0.6171; 0.5014–0.7327; *P* = 0.052), surgical history (0.616; 0.497–0.734; *P* = 0.056), symptom-to-admission interval (0.611; 0.4861–0.736; *P* = 0.066), and absence of pulmonary edema (0.602; 0.48–0.716; *P* = 0.091). Univariate logistic regression identified 11 significant predictors, excluding hormone use, which were subsequently included in multivariate analysis (**Table 2**; **Figure 1**).

**Table 2:** ROC curve and univariate binary logistic regression analysis of risk factors for PA.

Factors	ROC curve					Binary logistic regression analysis			
	Cutoff	Youden’s index	AUC	95% CI	P value	Coefficient	OR	95% CI	p value
Absence of Fever	NA	0.426	0.713	0.608-0.818	<0.001	1.936	6.93	2.62-18.34	<0.001
Viral coinfection	NA	0.331	0.665	0.551-0.780	0.006	1.589	4.9	1.93-12.43	<0.001
HB	111	0.392	0.6485	0.524-0.7731	0.014	1.792	6	2.39-15.04	<0.001
BDG	61.275	0.291	0.6249	0.5131-0.7367	0.038	2.273	9.71	2.54-37.11	<0.001
PCT	0.1045	0.206	0.6229	0.5086-0.7372	0.042	0.838	2.31	1.01-5.32	0.048
EO%	0.15	0.219	0.6171	0.5014-0.7327	0.052	0.971	2.64	1.11-6.27	0.028
Surgical history	NA	0.231	0.616	0.497-0.734	0.056	1.606	4.98	1.59-15.64	0.006
Symptom-to-admission interval	4.5	0.327	0.6111	0.4861-0.736	0.066	2.914	8.97	2.69-29.94	<0.001
Emphysema	NA	0.204	0.602	0.48-0.716	0.091	0.932	2.54	1.02-6.29	0.044
Hormone use	NA	0.175	0.587	0.469-0.705	0.149	0.836	2.308	0.942-5.651	0.067
Hematologic diseases	NA	0.124	0.562	0.442-0.682	0.304	1.676	5.34	1.02-28.04	0.048

PA, pulmonary aspergillosis; ROC Curve, receiver operating characteristic curves; AUC, area under the curve; CIs, confidence intervals; OR, odds ratio; HB, hemoglobin; BDG, (1, 3) -β -D-glucan; PCT, procalcitonin; EO%, eosinophil percent.

### 3.3 A diagnostic model for PA

**Figure 2:**
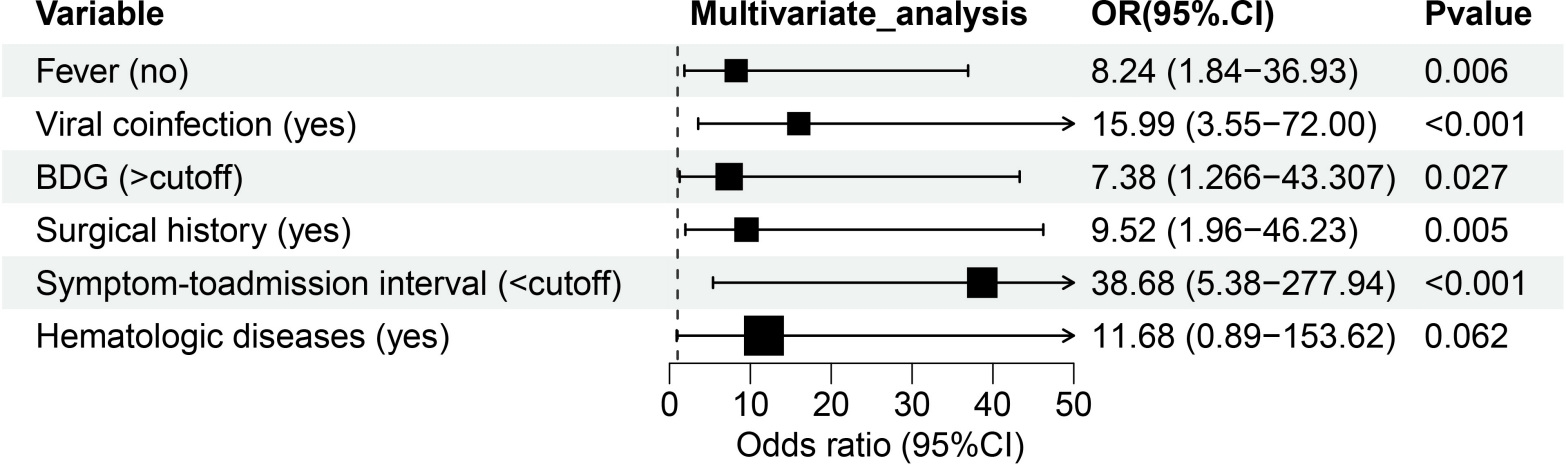
Forest map of 6 risk factors identified by the multivariate logistic analysis for the PA group. BDG, (1, 3) -β -D-glucan.

**Figure 3:**
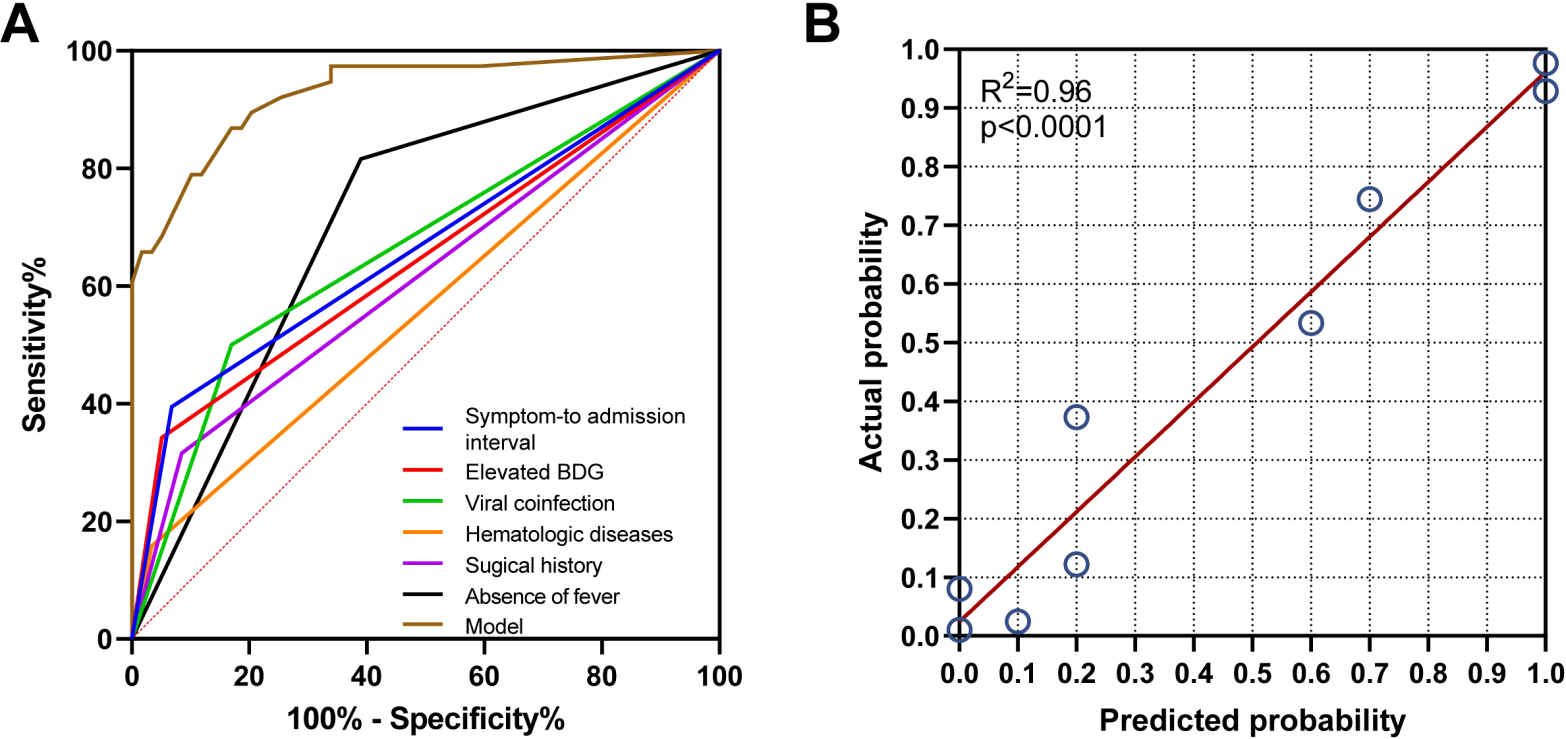
Validation of the model for predicting PA probability. **(A)** The receiver operating characteristic (ROC) curve analysis demonstrated the following area under the curve (AUC) values: model (0.93), absence of fever (0.713), surgical history (0.702), hematologic diseases (0.665), viral coinfection (0.616), (1,3)-β-D-glucan (BDG) (0.562), and symptom-to-admission interval (0.562). **(B)** The calibration plot demonstrated a strong agreement between the predicted probability of pulmonary aspergillosis (PA) and the actual observed outcome (R² = 0.96, p < 0.001).”.

As demonstrated in **Table 3**; **Figure 2**, multivariate analysis identified six significant predictors of PA: surgical history (OR: 9.52; 95% CI: 1.96–46.228; *P* = 0.005), hematologic diseases (OR: 11.678; 95% CI: 0.888–153.615; *P* = 0.062), absence of fever (OR: 8.244; 95% CI: 1.84–36.932; *P* = 0.006), viral coinfection (OR: 15.986; 95% CI: 3.549–72.002; *P* < 0.001), elevated BDG levels (> 0.6249 pg/mL; OR: 7.377; 95% CI: 1.256–43.307; *P* = 0.027), and shorter symptom-to-admission interval (< 4.5 days; OR: 38.681; 95% CI: 5.383–277.944; *P* < 0.001). The predictive model demonstrated excellent discrimination, with an AUC of 0.93 (95% CI: 0.877-0.984; *P* < 0.001; **Figure 3A**). Model was satisfactory, as evidenced by: a non-significant Hosmer-Lemeshow test (*P* = 0.606), strong agreement in the calibration plot (**Figure 3B**; R²=0.96, *P* < 0.001), a Brier score of 0.097 (95% CI: 0.0622-0.1382), indicating good overall predictive performance.

**Table 3:** Multivariate logistic regression analysis of risk factors for PA.

Risk factors	B	S. E	Wald χ^2^	p value	OR	95% CIs
Surgical history	2.253	0.806	7.811	0.005	9.52	1.96-46.228
Hematologic diseases	2.458	1.315	3.495	0.062	11.678	0.888-153.615
Absence of Fever	2.11	0.765	7.602	0.006	8.244	1.84-36.932
Viral coinfection	2.772	0.768	13.029	<0.001	15.986	3.549-72.002
BDG (>61.28 pg/mL)	1.998	0.903	4.896	0.027	7.377	1.256-43.307
Symptom-to-admission interval (< 4.5 days)	3.655	1.006	13.198	<0.001	38.681	5.383-277.944

PA, pulmonary aspergillosis; B coefficient; S.E. standard error; OR odds ratio; CIs confidence intervals; Wald, Wald χ^2^.

### 3.4 Diagnostic performance of mNGS for mixed infections

**Figure 4:**
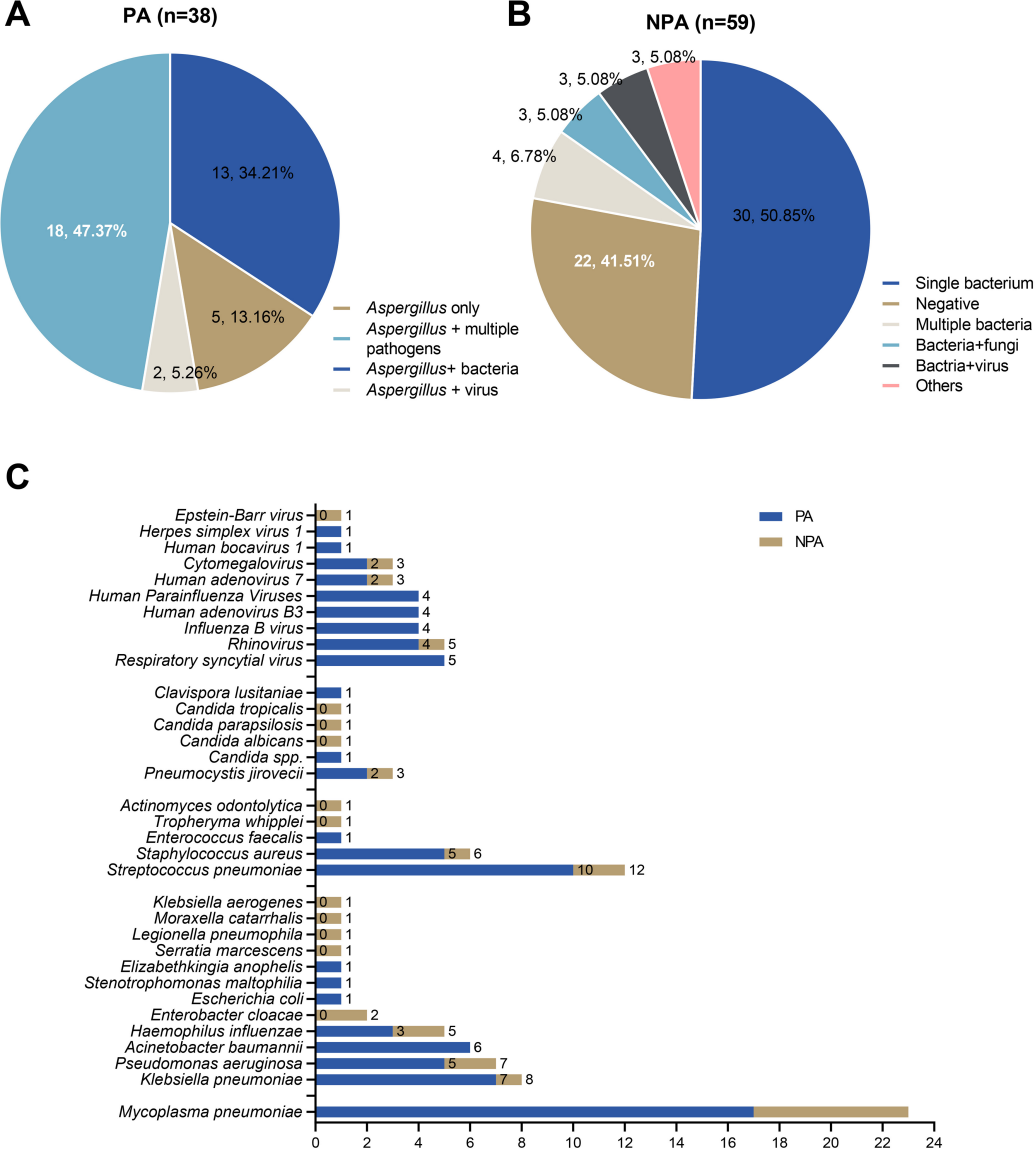
Mixed infections identified by mNGS in the PA and non-PA groups. **(A)** The mixed infections were identified by mNGS in the PA group. **(B)** The mixed infections were identified by mNGS in the non-PA group. **(C)** Comparative analysis of major co-pathogenic in PA group and non-PA group.

mNGS revealed significantly higher rates of polymicrobial infections in the PA group (86.84%) compared to non-PA group (11.54%; *P* < 0.001) (**Figures 4A, B**). The PA group predominantly exhibited coinfections with *Mycoplasma pneumoniae* and *Streptococcus pneumoniae* (**Figure 4C**). However, comparative analysis demonstrated no statistically significant differences in the co-infection patterns between PA and non-PA groups (**Figure 4C**).

## 4 Discussion

PA in pediatric populations remains a life-threatening infection with high morbidity and mortality, necessitating early and accurate diagnosis (Cadena et al., 2021; Olivier-Gougenheim et al., 2021; Jia et al., 2023). This study identified 6 independent risk factors for pediatric PA—surgical history, elevated serum BDG level, viral coinfection, absence of fever, shorter symptom-to-admission interval, and hematologic diseases—and established a predictive model with an AUC of 0.93. These discoveries redefined pediatric PA diagnosis by identifying child-specific biomarkers.

This study identified prior surgery as a key risk factor for pediatric PA (OR=9.52, 95% CI:1.96–46.23). This aligns with existing literature linking postoperative immunosuppression to increased susceptibility to invasive fungal infections in adults (Friol et al., 2025). However, this association between recent surgery and PA is stronger in children, likely due to immature immune regulation. Pediatric patients show impaired fungal clearance for the neutrophil dysfunction, with reduced reactive oxygen species (ROS) production and delayed apoptosis (Urban and Backman, 2020). Surgical stress exacerbates this by suppressing Th17-mediated immunity, essential for Aspergillus defense (Alexander et al., 2021). In our cohort, 60.53% of PA patients received corticosteroids, further weakening Th17 responses by inhibiting IL-23/IL-17 signaling (Gaffen et al., 2014). Surgical trauma combined with corticosteroid use creates an environment favorable to Aspergillus invasion (Lionakis et al., 2023).

In our predictive model, an elevated BDG level (cutoff: 61.275 pg/mL) demonstrated superior diagnostic performance over GM. This divergence from guideline recommendations (Warris et al., 2019); (Subspecialty Group of Respiratory, t.S.o.P.C.M.A et al., 2024) may be attributed to three pediatric-specific factors: 1) Antifungal Prophylaxis Impact: Triazole prophylaxis (e.g., posaconazole) in pediatric cohorts effectively suppresses Aspergillus galactomannan (GM) release by inhibiting fungal membrane synthesis, while BDG—a stable cell wall component—remains detectable (de Heer et al., 2019). This explains the predominantly negative serum GM results (<1.0 μg/L) in our study. 2) Pathogen Species Variation: Non-fumigatus Aspergillus species (e.g., *A. flavus, A. niger*) are more prevalent in children and produce lower GM levels than *A. fumigatus* (Hon et al., 2024). BDG, as a pan-fungal marker, is less affected by species-specific variations. 3) Age-Specific Diagnostic Thresholds: Our optimized BDG cutoff (61.275 pg/mL) exceeds the adult negative range (20–60 pg/mL), underscoring the necessity for pediatric-specific criteria—a gap highlighted in recent consensus guidelines (Lass-Florl et al., 2021). When used alone, BDG (>61.28 pg/mL) showed limited sensitivity (34.21%, 95% CI: 21.21–50.11%) but high specificity (94.92%, 95% CI: 86.08–98.61%). However, our composite model significantly improved sensitivity to 78.95% (95% CI: 63.65–88.93%) while maintaining specificity at 88.14% (95% CI: 77.48–94.13%) (**Supplementary Table 1**). This demonstrates the model’s capacity to integrate BDG with other predictors for enhanced pediatric PA detection. This aligns with findings that BDG sensitivity may surpass GM in specific clinical contexts (Dichtl et al., 2020).

This study identified a notable absence of fever in pediatric PA patients (61.02 vs. 18.42%, *P* < 0.001) compared to non-PA patients, highlighting atypical presentations in immunocompromised children (Wakai and Hess, 2023). Patients with PA do not exhibit specific fever symptoms compared to those infected with other pathogens (bacteria or viruses). This difference likely results from corticosteroid-mediated cytokine suppression (e.g., IL-6, TNF-α) and T-cell exhaustion, which attenuate febrile responses (Bjelakovic et al., 2010). These findings emphasize the need for increased clinical suspicion for PA in afebrile immunocompromised children, particularly as classic radiographic signs, such as the halo sign, are uncommon in pediatric cases (Thomas et al., 2019).

Hematologic diseases significantly increased the risk of pediatric PA (OR: 11.68, 95% CI: 0.89–153.62, P = 0.062), particularly in acute myeloid leukemia (AML; incidence: 3.7%) and hematopoietic stem cell transplantation (HSCT; incidence: 4.5%) (Niang et al., 2023; Wakai and Hess, 2023). Viral coinfection emerged as the strong risk factor (OR: 15.99, 95% CI: 3.55–72.00, P < 0.001), consistent with reports of influenza-associated (IAPA; prevalences: 15.3%) and COVID-19- (Wauters et al., 2021)associated pulmonary aspergillosis (CAPA, prevalences: 13%) in children (Wediasari et al., 2020; Ogawa et al., 2023; Li et al., 2024). Viruses may predispose to PA through airway epithelial damage and immune dysregulation, worsened by corticosteroid use (Costantini et al., 2020; Goncalves et al., 2024). Additionally, a shorter symptom-to-admission interval (OR: 38.68, 95% CI: 5.38–277.94, P < 0.001) indicates acute, severe presentations, potentially driven by viral coinfections or profound immunosuppression, as observed in invasive pulmonary aspergillosis (IPA) cases requiring urgent intervention (McMillan et al., 2020; Niang et al., 2023).

Our novel predictive model for pediatric PA achieves superior diagnostic accuracy (AUC: 0.93, R² = 0.96) compared to existing algorithms, which showed limited sensitivity (75-80%) (Niang et al., 2023; Wakai and Hess, 2023). The model’s enhanced discriminatory capacity appears to derive from incorporating two novel predictors: (1) viral coinfection and (2) symptom-to-admission interval. This marks a significant advancement over conventional models that relied primarily on neutropenia or radiographic findings (e.g., nodules or halo signs). Notably, the model demonstrates excellent calibration (Hosmer-Lemeshow p=0.34), indicating strong agreement between predicted and observed probabilities. These characteristics highlight its potential for clinical use, particularly for early identification of high-risk pediatric patients in critical care or immunocompromised settings.

mNGS revealed a high prevalence of polymicrobial infections in PA cases (86.84% vs. 18.64%, P < 0.001), highlighting the complexity of pediatric PA, particularly in viral-associated contexts. This finding aligns with reports of bacterial and viral co-pathogens in IAPA and CAPA (Ogawa et al., 2023; Li et al., 2024). mNGS proves valuable for detecting co-infections often missed by conventional culture or GM testing (Wakai and Hess, 2023). The polymicrobial burden may complicate antifungal therapy and worsen outcomes, necessitating tailored antimicrobial strategies.

This study has several limitations that warrant consideration. First, the study’s retrospective nature introduces potential selection bias, which may affect the generalizability of our findings. Second, the optimal BDG cutoff for pediatric populations remains uncertain due to ongoing debate regarding its diagnostic utility in children. And the lack of BALF GM data represents a key constraint, as serum GM (which was uniformly low in our cohort) is known to have lower sensitivity for PA compared to BALF testing. Third, the routine use of mNGS may be limited by its high cost and restricted accessibility, particularly in resource-limited settings. Our model’s performance requires validation in cohorts with complete GM testing (both serum and BALF) to assess its generalizability across different clinical settings. Multicenter prospective studies incorporating both BALF GM and standardized BDG testing are needed to: (a) establish pediatric-specific cutoff values, and (b) clarify the complementary roles of these biomarkers in different clinical scenarios (e.g., with/without antifungal prophylaxis).

## Conclusion

This study identifies critical risk factors and a high-performance predictive model for pediatric PA. The high polymicrobial rate revealed by mNGS emphasizes the need for comprehensive diagnostic approaches. These findings enable targeted risk stratification and early intervention, addressing a critical gap in pediatric PA management.

## Data Availability

The raw data supporting the conclusions of this article will be made available by the authors, without undue reservation.
