# Supplementary material for: Risk factors for identifying pulmonary aspergillosis in pediatric patients
Source: Front Cell Infect Microbiol. 2025 Jun 27;15:1616773. doi: 10.3389/fcimb.2025.1616773 (PMC12245894; doi:10.3389/fcimb.2025.1616773)
Supplement: Supplementary file 1 [file Table1.docx]

**Supplementary table 1. Sensitivity and specificity of risk factors for the diagnosis of PA**

| **Risk factors** | Sensitivity% | 95% CI | Specificity% | 95% CI |
| --- | --- | --- | --- | --- |
| Surgical history | 31.58 | 19.08% to 47.46% | 91.53 | 81.65% to 96.33% |
| Hematologic diseases | 15.79 | 7.444% to 30.42% | 96.61 | 88.46% to 99.40% |
| Absence of Fever | 81.58 | 66.58% to 90.78% | 61.02 | 48.27% to 72.42% |
| Viral coinfection | 50.00 | 34.85% to 65.15% | 83.05 | 71.54% to 90.52% |
| BDG (>61.28 pg/mL) | 34.21 | 21.21% to 50.11% | 94.92 | 86.08% to 98.61% |
| Symptom-to-admission interval (< 4.5 days) | 39.47 | 25.60% to 55.28% | 93.22 | 83.82% to 97.33% |
| Model (probability>0.5) | 78.95 | 63.65% to 88.93% | 88.14 | 77.48% to 94.13% |

Abbreviations: PA, pulmonary aspergillosis.
